# Giant left atrium causing severe dysphagia

**DOI:** 10.11604/pamj.2018.29.52.13607

**Published:** 2018-01-19

**Authors:** Attila Frigy

**Affiliations:** 1 Department of Internal Medicine IV, University of Medicine and Pharmacy of Tirgu Mures, Tirgu Mures, Romania

**Keywords:** Left atrium, esophageal stenosis, mitral prosthesis

## Image in medicine

A 65 years old men with mechanical mitral prosthesis (implanted for rheumatic valve disease) was referred to our clinic with signs of advanced heart failure and associated severe dysphagia. This symptom started a couple of years before and became worsened in the last months causing severe difficulties in alimentation, both for solid and liquid foods. Echocardiography revealed a normofunctional mitral prosthesis, a dilated left ventricle with severe systolic dysfunction (EF 15%) and a giant left atrium (A-parasternal long axis view, left atrial postero-anterior diameter = 80mm, B-apical four chamber view, left atrial longitudinal diameter = 105mm). The X-ray examination with barium of the upper gastrointestinal tract demonstrated significant lumen reduction in the middle and lower part of the esophagus (C), corresponding to the external compression caused by the huge left atrium. The explanations for the severely enlarged left atrium would be the late valvular intervention (also suggested by the dilated and dysfunctional left ventricle) and the progression of left atrial remodeling due to chronic inflammation. The patient was referred to esophageal balloon dilation and stent implantation.

**Figure 1:**
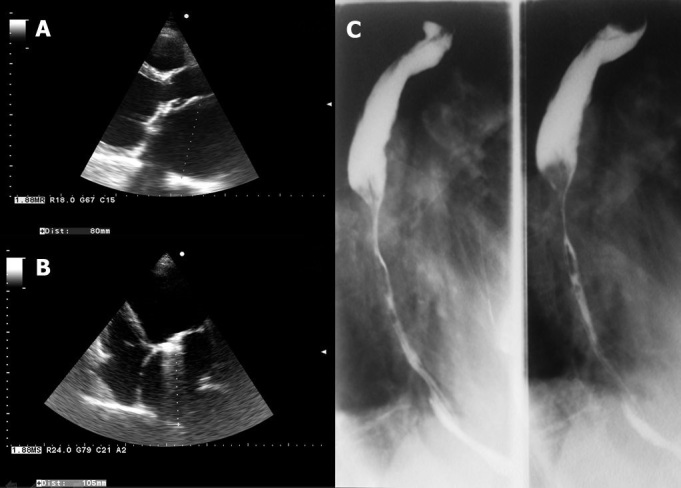
(A) echocardiography: parasternal long axis view, left atrial postero-anterior diameter = 80mm; (B) echocardiography: apical four chamber view, left atrial longitudinal diameter = 105mm); (C) X-ray examination with barium of the upper gastrointestinal tract demonstrating significant lumen reduction in the middle and lower part of the esophagus

